# Multiple calcifying fibrous tumors from gastric and retroperitoneal tissue: A case report

**DOI:** 10.1016/j.ijscr.2024.109944

**Published:** 2024-07-02

**Authors:** Masatsugu Ishii, Toshikatsu Nitta, Masataka Taki, Ryutaro Kubo, Atsushi Takeshita, Takashi Ishibashi

**Affiliations:** a Division of Surgery Gastroenterological Center, Medico Shunju Shiroyama Hospital, Habikino, Japan; b Department of Diagnostic Pathology, Osaka Medical and Pharmaceutical College Hospital, 2-7 Daigaku-machi, Takatsuki City, Osaka 569-8686, Japan

**Keywords:** Calcifying fibrous tumor, Gastritis, Lesser omentum, Case reports

## Abstract

**Introduction and importance:**

Calcifying fibrous tumor (CFT) is a rare benign mesenchymal lesion that has been occurred in the gastrointestinal tract, especially occurs most commonly in the stomach and the small and large intestines.

**Case presentation:**

74-year-old woman was admitted to our hospital with complaints of epigastric discomfort. Whole-body computed tomography (CT) revealed a 2.2-cm mass with a 1.2-cm low-density area at the anterior wall in the gastric cardia and lesser omentum; however, no abnormal uptake at the same site was noted on positron emission tomography (PET). We performed laparoscopic tumor resection of the two sites. The postoperative course was good without complications. Histopathological findings revealed collagen fibrous granulation connective tissue and psammomatous calcification. Subsequently, the patient has been relapse-free for 6 months.

**Clinical discussion:**

CFT is a rare benign tumor that commonly occurs in soft tissues, such as the subcutaneous extremities and neck. In particular, development from the stomach and lesser omentum has never been reported. Our case was incidentally found on a general examination. In our case, CT showed a low-density area, MRI showed a low-intensity area, and PET examination showed no uptake; it was difficult to establish a preoperative diagnosis. Therefore, in our case, laparoscopic tumor resection was performed, with GIST as the differential diagnosis.

**Conclusions:**

We herein reported a rare gastric and lesser omentum CFT that was successfully treated by laparoscopic surgery. For curative treatment of CFT, complete surgical resection is necessary.

## Highlights

• Multiple calcifying fibrous tumors from gastric and retroperitoneal tissue are rare and has been detected incidentally on a routine imaging examination.
• It is difficult to differentiate from mesenchymal tumors such as gastrointestinal submucosal tumor (GIST) as a preoperative diagnosis.
• Complete laparoscopic surgical excision is the effective treatment for CFT removing the risk of recurrence or metastasis.

## 1 Introduction

Calcifying fibrous tumors (CFT) are rare benign tumors that usually develop from soft tissues and reportedly develop in the pleura, peritoneum, lungs, mediastinum, stomach, adrenal gland, paratesticular organs, and spermatic cord [[1], [2], [3], [4], [5], [6]].

The development of CFT from the stomach in the gastrointestinal tract is relatively rare and difficult to differentiate from mesenchymal tumors such as gastrointestinal submucosal tumor (GIST) as a preoperative diagnosis. Complete surgical excision is the typical treatment for CFT.

Here we report a case of the laparoscopic resection of two CFT arising from the stomach and lesser omentum. This work has been reported in line with the SCARE criteria [11].

## 2 Case presentation

### 2.1 Current medical history

**Fig. 1:**
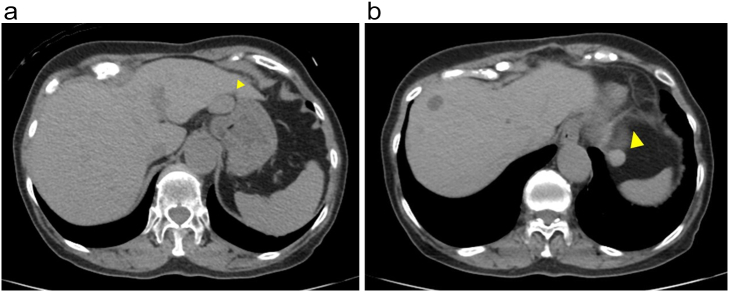
Abdominal CT. Ischemic lesions approximately 22 mm and 12 mm in the lower portion of the stomach and superior lobe of the spleen, respectively (yellow arrow).

**Figs. 2,3:**
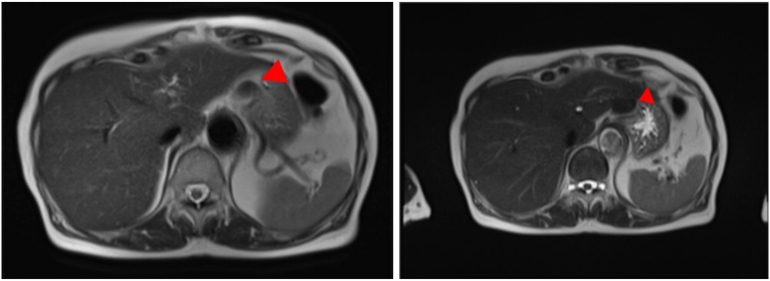

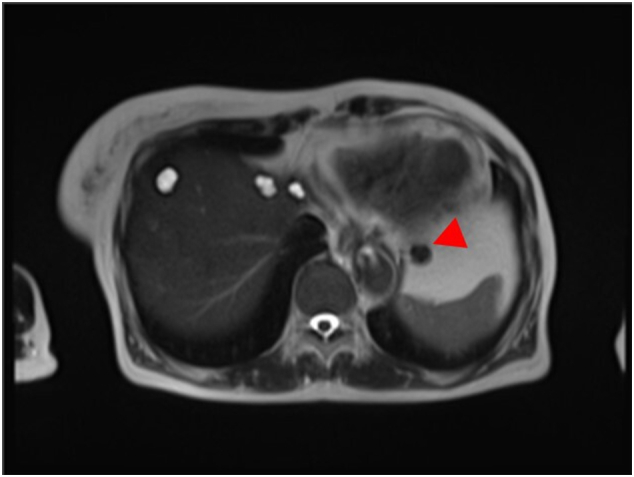
Abdominal MRI. Two space-occupying lesions, approximately 22 mm and 12 mm in diameter, that were low-intensity areas on T2-weighted images, were observed on the caudal side of the left lobe of the liver and the superior part of the spleen (red arrow).

In January 2021, 74-year-old woman was admitted to our hospital with complaints of epigastric discomfort. The blood examination findings were within normal ranges. An intra-abdominal mass was detected on abdominal ultrasound during a general screening examination, preoperative serum CEA level was normal (3.9 mg/dL) in blood examination findings. Abdominal CT revealed ischemic lesions approximately 22 mm and 12 mm in the lower portion of the stomach and superior lobe of the spleen, respectively (Fig. 1, yellow arrow). MRI showed two space-occupying lesions, approximately 22 mm and 12 mm in diameter, that were low-intensity areas on T2-weighted images, were observed on the caudal side of the left lobe of the liver and the superior part of the spleen (Figs. 2,3, red arrow). And in PET-CT examination, no accumulation was noted consistent with the mass of the lesser curvature of the stomach and the superior lobe the spleen.

Laparoscopic resection was performed. No lymph node metastasis, distant metastasis, or infiltration into other organs was observed macroscopically, including on preoperative imaging. The tumor originated from the anterior wall of the lesser curvature of the stomach and the anterior wall of the gastric fornix.

**Fig. 4:**
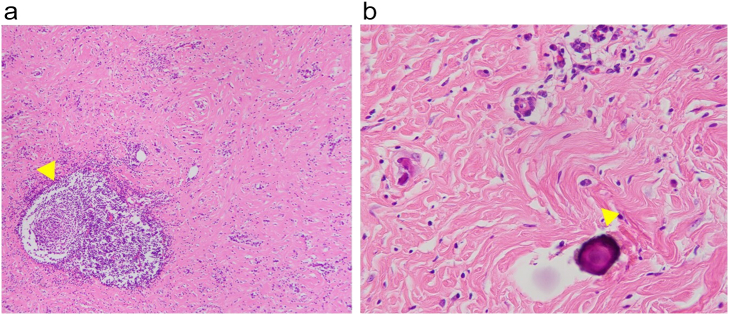
Histopathological findings. Collagen fibrous granulation connective tissue and psammomatous calcification (yellow arrow) spread into multiple nodules accompanied by lymphocytic infiltration and lymphoid folliculogenesis (yellow arrow).

Histopathological findings demonstrated collagen fibrous granulation connective tissue and psammomatous calcification spread into multiple nodules accompanied by lymphocytic infiltration and lymphoid folliculogenesis (Fig. 4, yellow arrow); the histopathological findings are consistent with the diagnosis; the findings were not diagnosed as. The postoperative course was good without complications, and the patient was discharged on the 9th postoperative day. The patient remains well with no evidence of tumor recurrence for 6 months after resection.

## 3 Discussion

CFT is a rare benign tumor that commonly occurs in soft tissues, such as the subcutaneous extremities and neck. CFT was first reported by Rosenthal and Abdul in 1988 as childhood fibrous tumor with psammoma bodies [7]. Other organs such as the pleura, lung, mediastinum, peritoneum, adrenal gland, paratesticular organs, and spermatic cord are the primary sites [[1], [2], [3], [4], [5], [6], [7]]. In particular, a search of *Japanese Journal of Medicine* (1983–2022) using keywords “calcified fibrous tumor” or “calcified fibrous pseudotumor” (excluding conference proceedings) retrieved only six cases of CFT occurring in the stomach [8]. Moreover, development from the stomach and lesser omentum has never been reported. Our case was incidentally found on a general examination, but intussusception in the small intestine may also be triggered by intussusception [9].

Generally, CT and MRI examinations are performed for preoperative diagnosis. However, CT often reveals punctate and linear calcifications. Furthermore, it is often difficult to differentiate these tumors from malignant tumors, and PET is sometimes used in combination.

In our case, laparoscopic tumor resection was performed, with GIST as the differential diagnosis. Intraoperative findings included tumors originating from the lesser omentum and the anterior wall of the gastric fornix, and two tumors were excised under arthroscopic guidance. Compared with the six other reported cases, our case did not show a clear submucosal tumor-like morphology on upper gastrointestinal endoscopy, and the tumor did not have a capsule.

A search of *Japan Journal of Medicine* using the keywords “stomach” and “calcifying fibrous tumor” (1964–2022) revealed only seven cases of gastric primary CFT including ours. There were no co-occurrences of preoperative differential diagnoses, including GIST, leiomyoma, schwannoma, and immunoglobulin G4–related malignant mesothelioma. In our case, CT showed a low-density area, MRI showed a low-intensity area, and PET examination showed no uptake; therefore, it was difficult to establish a preoperative diagnosis.

The causes and mechanisms underlying CFT remain unknown. Generally, the pathological findings include spindle-shaped cells, hyperplasia of plasma cells, and scattered calcifications within the tumor. Its treatment generally involves complete resection of the tumor [[12], [13], [14], [15]].

In our case, we considered two types of tumors during the preoperative examination: extraductal gastric GIST, leiomyoma, and solitary tumors. The tumors were 2.2 cm and 1.2 cm under laparoscopic examination. A tumor originating from the anterior wall of the gastric vault exhibited extraductal growth for which a partial gastrectomy was performed. The tumor originating from the lesser omentum showed mild infiltration into the surrounding tissues and organs, and parts of the lesser omentum and the tumor were resected. Using a laparoscope, it was possible to observe in detail the tumor site and infiltration degree in the abdominal cavity. The prognosis of CFT is good, and Chorti et al. [10] reported recurrence in 10 of 96 patients at a mean 29 months postoperative. In our case, the patient was alive without recurrence 6 months after surgery. Due to the rarity of this disease, consensus is lacking regarding the long-term prognosis of CFT in various organs.

## 4 Conclusions

We herein reported a rare gastric and lesser omentum CFT that was successfully treated by laparoscopic surgery. For curative treatment of CFT, complete surgical resection is necessary.

## Ethical approval

This article is in accordance with the Declaration of Helsinki.

The study was conducted according to the relevant guideline and regulations, and the institutional review board of the Shiroyama Hospital. (APPROVAL NUMBER/2018-004).

## Funding

The authors (s) received no financial support for the research, authorship, or publication of this article.

## Author contribution

MI carried out the acquisition of data and drafted the manuscript. MT, RK, and TI were involved in the drafting of the manuscript. AT performed immunohistochemical staining. TN critically revised the manuscript. All authors have read and approved the final manuscript.

## Guarantor

Dr. Toshikatsu Nitta

## Research registration number

Not applicable.

## Consent

Verbal informed consent was obtained from legally authorized representatives for the publication of anonymized patient information in this article. Written informed consent was obtained from the patient for publication and any accompanying images. A copy of the written consent is available for review by the Editor-in-Chief of this journal on request.

## Conflict of interest statement

The authors declared no conflict of interest.
